# Overexpression of MAL2 Correlates with Immune Infiltration and Poor Prognosis in Breast Cancer

**DOI:** 10.1155/2021/5557873

**Published:** 2021-09-09

**Authors:** Yue Zhong, Zhenjie Zhuang, PeiJu Mo, Qi Shang, Mandi Lin, JiaQian Gong, JiaRong Huang, HaiYan Mo, Mei Huang

**Affiliations:** ^1^Guangzhou University of Chinese Medicine, Guangzhou, Guangdong, China; ^2^Department of Breast Diseases, The First Affiliated Hospital of Guangzhou University of Chinese Medicine, Guangzhou, Guangdong, China

## Abstract

**Background:**

Myelin and lymphocyte, T cell differentiation protein 2 (MAL2) is highly expressed in various cancers and associated with the development and prognosis of cancer. However, the relationship between MAL2 and breast cancer requires further investigation. This study aimed to explore the prognostic significance of MAL2 in breast cancer.

**Methods:**

MAL2 expression was initially assessed using the Oncomine database and The Cancer Genome Atlas (TCGA) database and verified by quantitative real-time polymerase chain reaction (RT-qPCR). The chi-square test or Fisher's exact test was used to explore the association between clinical characteristics and MAL2 expression. The prognostic value of MAL2 in breast cancer was assessed by the Kaplan–Meier method and Cox regression analysis. Gene set enrichment analysis (GSEA) was performed to identify the biological pathways correlated with MAL2 expression in breast cancer. Besides, a single-sample GSEA (ssGSEA) was used to assess the relationship between the level of immune infiltration and MAL2 in breast cancer.

**Results:**

Both bioinformatics and RT-qPCR results showed that MAL2 was expressed at high levels in breast cancer tissues compared with the adjacent tissues. The chi-square test or Fisher's exact test indicated that MAL2 expression was related to stage, *M* classification, and vital status. Kaplan–Meier curves implicated that high MAL2 expression was significantly associated with the poor prognosis. Cox regression models showed that high MAL2 expression could be an independent risk factor for breast cancer. GSEA showed that 14 signaling pathways were enriched in the high-MAL2-expression group. Besides, the MAL2 expression level negatively correlated with infiltrating levels of eosinophils and plasmacytoid dendritic cells in breast cancer.

**Conclusion:**

Overexpression of MAL2 correlates with poor prognosis and lower immune infiltrating levels of eosinophils and plasmacytoid dendritic cells in breast cancer and may become a biomarker for breast cancer prognosis.

## 1. Introduction

Among women, breast cancer is the most commonly diagnosed cancer and the main cause of cancer death [1]. The incidence of breast cancer has increased every year [2]. Nearly 3.8 million women in the United States have been diagnosed with breast cancer, including 268,600 new cases in 2019 [3].

Despite the advances in surgery, chemotherapy, radiotherapy, and endocrine therapy for breast cancer, more than 20% of patients still develop metastatic disease with a poor prognosis [4]. Since the molecular mechanisms of breast cancer remain unclear, the identification of novel prognostic biomarkers for breast cancer is necessary, which could contribute to the early detection and treatment of breast cancer [5]. MAL2, as an essential member of the MAL proteolipid family, is a four-pass membrane protein consisting of 176 amino acid residues. By encoding a multi-transmembrane protein, MAL2 mainly participates in endocytosis under physiological conditions and mediates the transport of substances between cells [6]. MAL2 is located in chromosome 8q23, an area where the copy number often increases in various types of cancer [7]. Previous studies have confirmed the increased expression of MAL2 in ovarian cancer [6], pancreatic cancer [8], thyroid cancer [9], and colorectal cancer [10]. Moreover, MAL2 expression was associated with pancreatic cancer and colorectal cancer overall survival [8], which suggested that MAL2 might be an important molecule involved in the progression and prognosis of tumors. Besides, MAL2 was recently identified as a breast cancer immunology target. Reduction of MAL2 in breast tumor cells can enhance CD8+ T cell-mediated cytotoxicity and inhibit the growth of breast tumors [11]. Although some previous studies suggested that MAL2 was overexpressed in breast cancer [7, 12, 13], the prognostic value of the MAL2 expression and its correlation with clinical features and immunotherapy in breast cancer requires further investigation, and whether MAL2 could be a specific marker for breast cancer still needs to be elucidated. In the present study, we applied a comprehensive strategy to uncover the importance of MAL2 in breast cancer.

## 2. Materials and Methods

### 2.1. Oncomine Database

The Oncomine database [14] (http://www.oncomine.org), a gene chip-based database and data mining platform, served to analyze the expression of MAL2 in various types of cancers. The filter conditions set were as follows: gene, MAL2; cancer type, breast cancer; differential analysis, cancer vs normal analysis; and data type, mRNA. Besides, we selected *P* value = 1E–4, twofold change, and top 10% gene rank as the threshold [15]. All statistical methods and results were obtained from Oncomine.

### 2.2. The Cancer Genome Atlas

The Cancer Genome Atlas (TCGA) is a landmark cancer genomics program that comprised over 20,000 primary cancer data and matched normal samples spanning 33 cancer types. Raw counts of RNA-sequencing data and corresponding clinical information were obtained from The Cancer Genome Atlas (TCGA) data set (https://www.cancer.gov/tcga).

### 2.3. The Verification of MAL2 by qRT-PCR

Tumor tissues and paired adjacent tissues were collected from patients diagnosed with breast cancer at the First Affiliated Hospital of Guangzhou University of Chinese Medicine between 2019 and 2020. None of these patients received therapy before surgery. Specimens from surgery were stored at −80°C. Study approval was obtained from the Research Ethics Committee of the First Affiliated Hospital of Guangzhou University of Chinese Medicine, and patients signed informed consent forms before operation.

Total RNA was extracted from 32 pairs of breast cancer tissues and adjacent nontumor tissues frozen in liquid nitrogen using TRIzol reagent (Invitrogen, United States). The isolated RNA was measured at 260/280 nm using a NanoDrop 2000 spectrophotometer (Thermo Fisher Scientific, USA). Evo M-MLV RT Premix for qPCR (AG11706, China) was used to reverse-transcribe RNA into cDNA. According to the manufacturer's instructions, cDNAs were amplified using the SYBR Green Premix Pro Taq HS qPCR Kit (AG11701, China). *β*-actin was used as an internal control for mRNA expression. The primer sequences were as follows: MAL2 Forward: 5′-ACGTAGCAGCCTCAATTTTTGC-3′ and Reverse: 5′-CATCTTCGTAAAGCCAGACCC-3′; *β*-actin, forward: 5′-TGGCACCCAGCACAATGAA-3′ and reverse: 5′-CTAAGTCATAGTCCGCCTAGAAGCA-3′. Each sample was carried out three times, and data were calculated using the 2^−ΔΔCt^ (2 to the power of minus Delta Delta CT) method.

### 2.4. Statistical Analyses

Data analysis was performed using SPSS software 26.0 (IBM Corporation, Armonk, NY, USA) and R version 3.6.3 [16]. The ggplot2 package [17] was used to draw boxplots of clinical features according to MAL2 expression variation. The chi-square test or Fisher's exact test was used to explore the association between clinical characteristics and MAL2 expression. The Wilcoxon signed rank sum test and Kruskal–Wallis test were utilized to measure the differential expression of MAL2 in the subgroup, including age; tumor (T), node (N), and metastasis (M) classification; estrogen receptor (ER) status; progesterone receptor (PR) status; human epidermal growth factor receptor-2 (HER2) status; and vital status. The impact of MAL2 expression levels on the overall survival (OS) of patients with breast cancer was analyzed by Kaplan–Meier curves using an R package named survminer [16].

Since we aimed to analyze the relationship of MAL2 with clinical features and its significance on the survival of patients with breast cancer, the median expression value of MAL2 was applied as a cut-off value for further analysis according to the previous studies [18–21]. Therefore, the median value of MAL2 expression was utilized as a cut-off value to divide patients with complete clinical data from the TCGA database into the high-MAL2-expression group and low-MAL2-expression group.

Then, the univariate and multivariate Cox analyses were performed to determine the related variables.

### 2.5. Gene Set Enrichment Analysis

Gene set enrichment analysis (GSEA) [22] was conducted to determine the biological processes activated in the low-MAL2-expression and high-MAL2-expression groups. GSEA software (version 4.0) was downloaded from their official website. Tumor tissue samples were divided into high-expression groups and low-expression groups according to the median value of MAL2 expression level. The gene set of h.all.v7.4.symbols.gmt and c2.cp.kegg.v7.4.symbols.gmt, containing a large number of tumors signaling pathway gene sets, were obtained from the MSigDB database. Depending on the default parameters of GSEA software, 1,000 times a random combination was used for enrichment analysis. Sorted by false discovery rates (FDRs), the gene set with FDR <0.25 and normal *P* value <0.05 was the significantly enriched gene set [22].

### 2.6. Immune Infiltration Analysis of MAL2

The transcripts per million (TPM) normalized RNA-seq data of breast cancer were downloaded from UCSC Xena (https://xena.ucsc.edu/) [23]. Twenty-eight immune gene sets, including different classic immune cell types, were obtained from the study by Charoentong et al. [24]. Next, the ssGSEA was performed by R package GSVA [25] to assess the immune infiltration level of each sample of breast cancer and calculate the responding immune infiltration score based on the TPM data and gene sets [25]. Subsequently, Spearman's correlation analysis between the MAL2 expression data and the immune infiltration score of 28 immune gene sets was performed. The threshold was set as *P* value < 0.05 and |r| ≥ 0.3 to filter the immune cell types that have significantly infiltrating level correlation with the MAL2 expression.

## 3. Results

### 3.1. Overexpression of MAL2 in Breast Cancer Based on Oncomine

**Figure 1:**
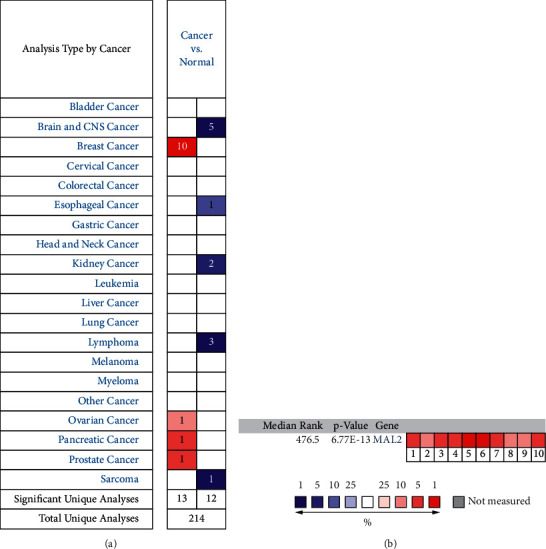
MAL2 expression in different types of cancers. (a) Expression of MAL2 gene in various cancers compared with matched normal tissues by the Oncomine database. Red and blue represent the number of data sets of increasing and decreasing MAL2 gene levels, respectively. (b) A meta-analysis of MAL2 expression across 10 analyses from the Oncomine database. Curtis breast (1–3), Ma breast (4), Perou breast (5), Sorlie breast (6–7), and TCGA breast (8–10). The colored squares represent the median rank of these genes (tumor tissues vs. normal tissues) across the 10 data sets. The significance level for the median rank analysis was set at *P* < 0.05.

We used Oncomine to analyze the difference between MAL2 tumor and normal tissues in different cancer types. Altogether, 214 different types of research results were collected in the Oncomine database. Among them, 25 results showed statistically different expressions of MAL2, including 13 with increased expression and 12 with decreased expression. Results revealed that the MAL2 expression level increased in breast, ovarian, prostate, and pancreatic cancers. On the contrary, MAL2 expression decreased in some cancer types, such as sarcoma, brain and CNS, esophageal, kidney, and lymphoma cancer (Figure 1(a)) [14]. To assess further the MAL2 expression in breast cancer, we analyzed MAL2 across six public expressions, containing 10 analyses from the Oncomine database. As a result, MAL2 was found to significantly upregulate in breast carcinoma tissues. Details are shown in Table 1. Based on the results of the meta-analysis of six data sets, including 10 analyses using the Oncomine database, the results indicated MAL2 overexpression in tumor tissues (median rank = 476.5, *P*=6.77E − 13) (Figure 1(b)).

### 3.2. MAL2 Was Upregulated in Breast Cancer Tissues Compared with Adjacent Tissues Based on TCGA

**Figure 2:**
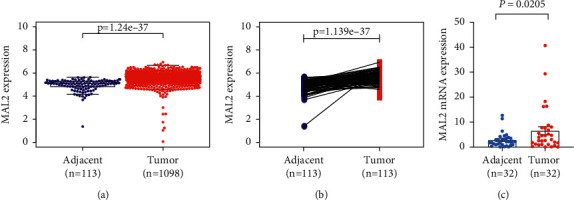
Different MAL2 mRNA expression in tumor tissues compared with adjacent tissues isolated from breast cancer patients. (a) MAL2 mRNA expression was significantly higher in tumor tissues than in adjacent tissues. (b) Paired breast cancer patient samples revealed that MAL2 expression was also higher in tumor tissues than in paired adjacent tissues. (c) RT-qPCR analysis of MAL2 mRNA expression in 32 pairs of breast cancer tissues and adjacent tissues.

The MAL2 expression data of 1098 breast cancer samples and 113 adjacent controls were retrieved from the TCGA database. We measured the differences in MAL2 expression in tumor and adjacent tissues using the independent-sample Wilcoxon rank sum test and the paired Wilcoxon rank sum test. Results showed that MAL2 expression was significantly higher in different tissues (tumor tissues vs. adjacent tissues, *P*=1.24*e* − 37, Figure 2(a); paired tumor vs. adjacent tissues, *P*=1.139*e* − 37, Figure 2(b)).

### 3.3. Verification of MAL2 Upregulation in Breast Cancer by RT-qPCR

To certify the difference in MAL2 expression in the TCGA data set, we performed RT-qPCR to detect the expression of MAL2 in 32 pairs of breast cancer tissues (including 32 tumor tissues and 32 adjacent tissues). The result showed that MAL2 mRNA was significantly increased in breast cancer tumors compared with adjacent tissues (*P*=0.0205, Figure 2(c)), which was consistent with the Oncomine data sets analyses.

### 3.4. Association of MAL2 Expression with Clinical Features in Breast Cancer

**Figure 3:**
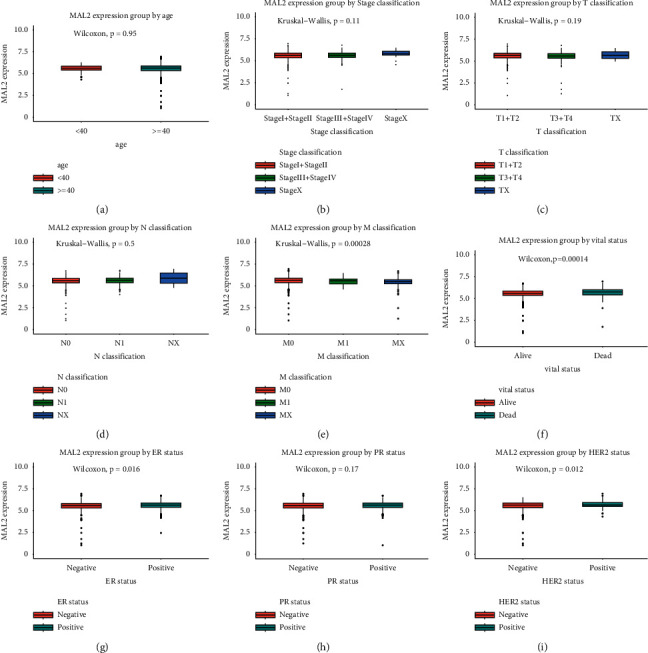
Differential MAL2 expressions in the boxplot. The expression of MAL2 is grouped by age (a), stage (b), *T* classification (c), *N* classification (d), *M* classification (e), vital status (f), ER status (g), PR status (h), and HER2 status (i).

We downloaded mRNA-seq data and clinical information of breast cancer patients from TCGA. There were 1211 cases of mRNA-seq data (1098 tumor samples; 113 adjacent tissues) derived from 1091 breast cancer patients in the TCGA database of 1090 equipped with complete clinical data. 1090 patients were finally enrolled in the study. The detailed clinical characteristics—age, gender, *T*, *N*, *M* classification, stage, ER, PR, HER2 status, vital status, and MAL2 expression—are presented in Table 2. In Figure 3, boxplots presented the MAL2 was expressed with the significant difference in the subgroup by *M* classification (*P*=0.0028), ER status (*P*=0.016), HER2 status (*P*=0.012), and vital status (*P*=0.00014).

### 3.5. Relationship between MAL2 Expression and Clinical Features in Breast Cancer

To determine correlations of MAL2 expression with clinical factors, we conducted a chi-square test. The median value of MAL2 expression was used to divide patients into a MAL2 high-expression group and a low-expression group. We observed that MAL2 expression was significantly associated with stage (*P*=0.044), M classification (*P*=0.002), and vital status (*P*=0.003) (Table 3).

### 3.6. MAL2 Overexpression Independently Predicted Poor Overall Survival

**Figure 4:**
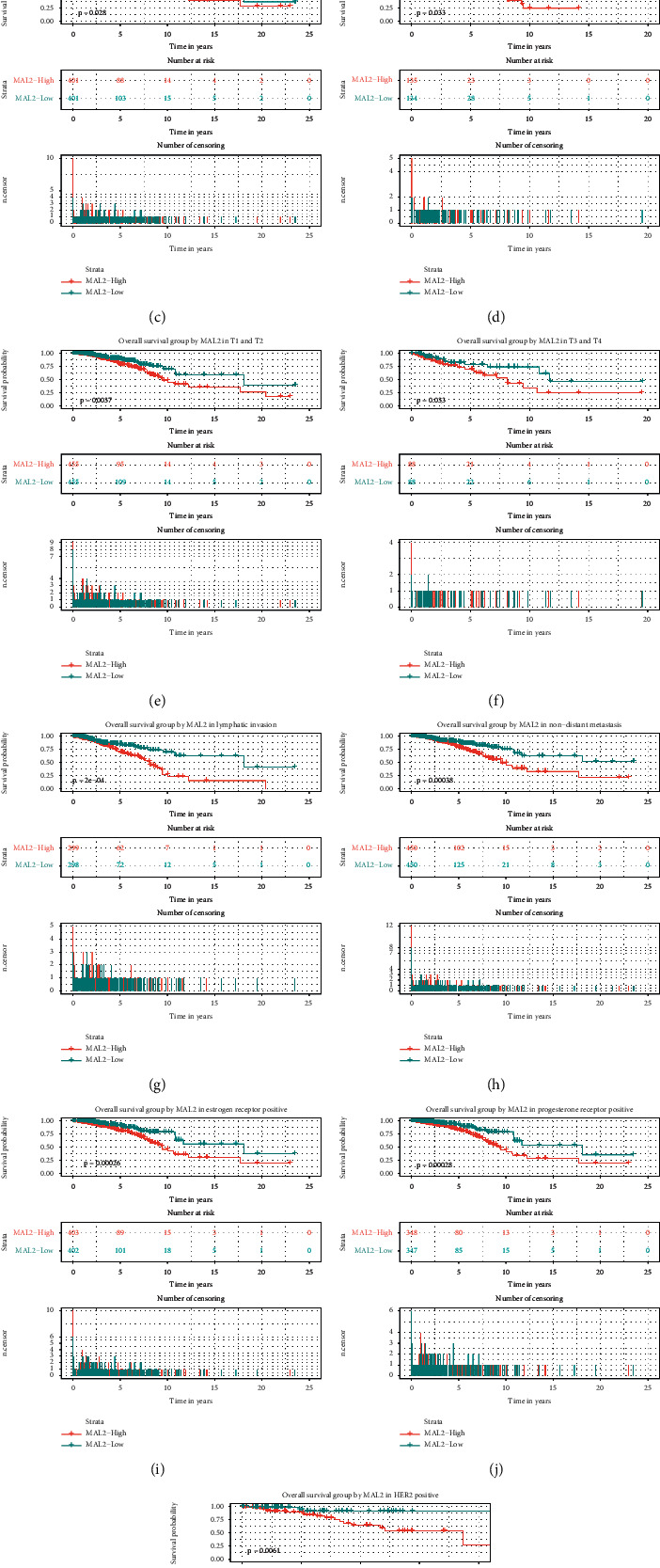
Survival analysis of MAL2 expression in terms of overall survival. Kaplan—Meier survival curve analysis of all tumors (a), subgroup analysis of old patients (b), clinical stage I/II and III/IV (c and d), subgroup analysis of *T* classification (*T*1/*T*2 and *T*3/*T*4) (e and f), patients with lymphatic invasion (g), patients with nondistant metastasis (h), and patients with ER-, PR-, and HER2-positive status (i—k, respectively).

Kaplan–Meier curves with the log-rank test were applied to explore the prognostic value of MAL2 expression and the overall breast cancer survival rate. As shown in Figure 4, breast cancer patients with high MAL2 expression were associated with worse overall survival (*P*=0.00093). In addition, high MAL2 expression was associated with poor overall survival in old patients (*P*=0.0015); clinical stage I/II and III/IV (*P*=0.028 and *P*=0.033); subgroup analysis of *T* classification (T1/T2 and T3/T4) (*P*=0.037 and *P*=0.033); patients with lymphatic invasion (*P*=0.0002); patients with nondistant metastasis (*P*=0.00038); and patients with positive ER, PR, and HER2 status (*P*=0.00026, *P*=0.00028, and *P*=0.0061, respectively).

**Figure 5:**
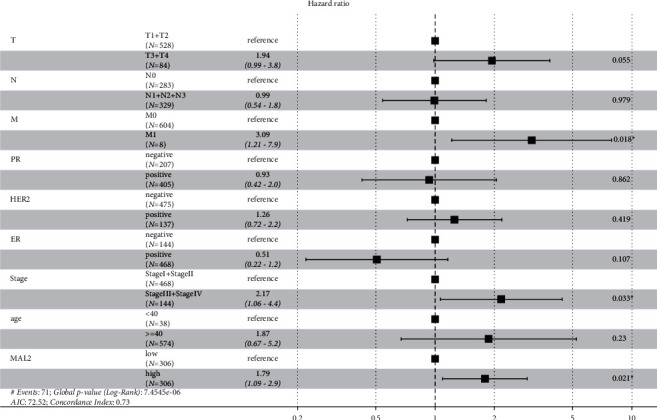
Forest plot for Cox proportional hazards model of overall survival in breast cancer.

The univariate Cox proportional hazards model showed that MAL2 expression, stage, *T* classification, *N* classification, and *M* classification of breast cancer patients were significantly correlated with the prognosis of patients. Moreover, the multivariate Cox proportional hazards model indicated that high MAL2 expression (hazard ratio = 1.792, *P*=0.021), stage (hazard ratio = 1.473, *P*=0.033), and *M* classification (hazard ratio = 3.093, *P*=0.0018) were independent risk factors for overall survival. The median overall survival of the high-expression group was 9.5 years, while that of the low-expression group was 18 years. Both the log-rank test and Cox proportional hazards model showed that the expression of MAL2 was significantly correlated with the prognosis of breast cancer. These results are described in Table 4 and Figure 5.

In conclusion, both in the Kaplan–Meier model and the Cox proportional hazard regression model, the results indicated that MAL2 expression in breast cancer was significantly correlated with the prognosis of patients.

### 3.7. Identification of MAL2-Related Signaling Pathways by GSEA

**Figure 6:**
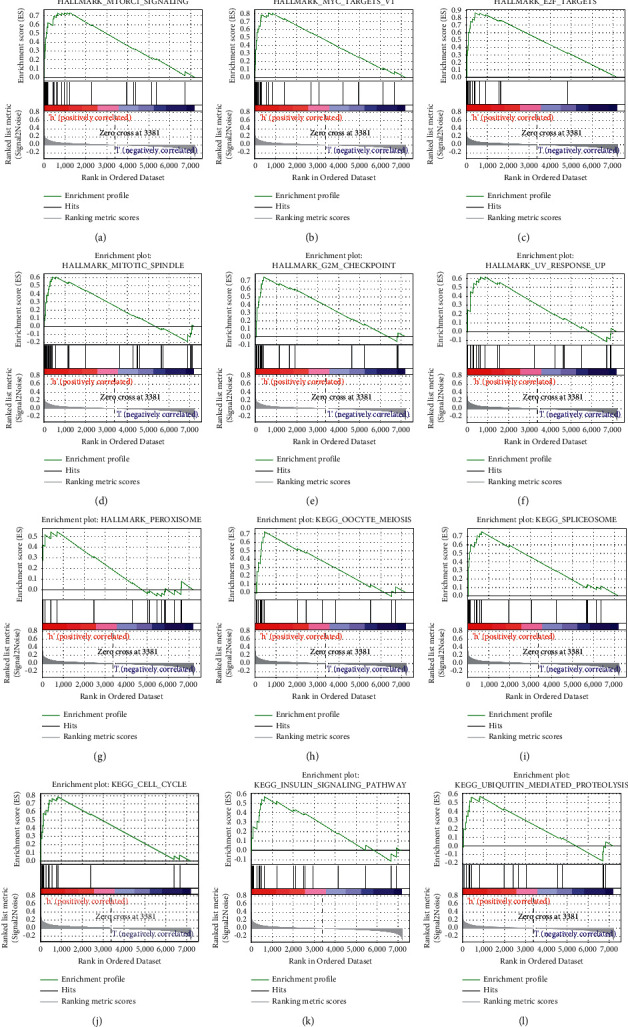
Enrichment plots of GSEA in breast cancer with a high-MAL2-expression phenotype. GSEA results showed that MYC targets V1 (a), mTORC1 signaling pathway (b), E2F targets (c), mitotic spindle (d), G2M checkpoint (e), UA response (f), peroxisome (g), oocyte meiosis (h), spliceosome (i), cell cycle (j), insulin signaling pathway (k), and ubiquitin-mediated proteolysis (l) were enriched in high MAL2 expression in breast cancer.

Data sets from GSEA showed significant differences (|NES| > 1, FDR < 0.25, NOM *P* < 0.05) in MSigDB Collection. The details are described in Figure 6 and Table 5.

The significant pathways by GSEA included MYC targets V1, mTORC1 signaling pathway, insulin signaling pathway, E2F targets, UA response, G2M checkpoint, oocyte meiosis, mitotic spindle, peroxisome, spliceosome, cell cycle, and ubiquitin-mediated proteolysis enriched differentially in MAL2 high-expression phenotype.

### 3.8. High-Expressed MAL2 Correlates with Reduced Immune Infiltration in Breast Cancer

**Figure 7:**
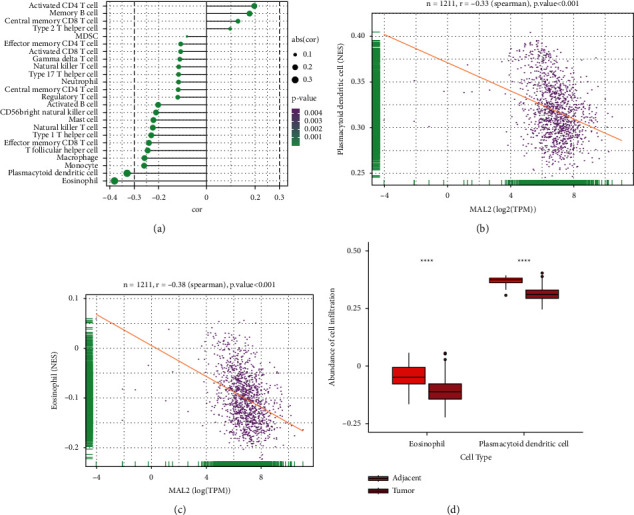
Association analysis of MAL2 gene expression and immune infiltration: (a) association analysis between MAL2 expression and immune cells; (b) association analysis of MAL2 expression with immune infiltration levels of eosinophils; (c) association analysis of MAL2 expression with immune infiltration levels of plasmacytoid dendritic cells; and (d) abundance of cell infiltration of eosinophils and plasmacytoid dendritic cells in breast cancer.

By Spearman's correlation test, the MAL2 expression level was found to be negatively correlated with infiltrating levels of eosinophils (*r* = −0.38, *P* < 0.01) and plasmacytoid dendritic cells (*r* = −0.33, *P* < 0.01) (Figures 7(a)–7(c)). Additionally, the abundance of infiltration of both eosinophils and plasmacytoid dendritic cells was significantly lower in tumor tissues than in adjacent tissues (Figure 7(d)).

## 4. Discussion

Breast cancer is the dominating cause of cancer-related mortality among females worldwide. With the rapid development of genomics and molecular biology, identifying the crucial biomarkers for the diagnosis and treatment of breast cancer has become an important research tendency. Recently, numerous research studies showed that MAL2 could be a promising biomarker in various solid tumors. Jennifer et al. showed that MAL2 was increased in ovarian carcinoma, and the overexpression of D52, the binding partner of MAL2, was linked to low overall survival in breast cancer [13]. Chen et al. found that MAL2 was utilized to distinguish pancreatic ductal adenocarcinoma from chronic pancreatitis [26]. Also, the high expression of MAL2 contributed to the short survival time and high distant metastasis rate of postoperative pancreatic cancer patients [8]. Besides, it was reported that MAL2 expression increased in colon cancer tissues and lymph nodes with metastasis with high accuracy and specificity in the diagnosis of colon cancer [10].

As for breast cancer, previous studies showed that MAL2 could induce proliferation and invasion of breast cancer cell lines by adjusting the epithelial-mesenchymal transition [12]. MAL2 was also proved to promote immune evasion by suppressing tumor antigen presentation in breast cancer [11]. However, a further study about the correlation between MAL2 and breast cancer was needed. Here, we conducted this study to consider further the significance of MAL2 expression in the prognosis of breast cancer.

Our study proved the value of MAL2 in breast cancer. MAL2 was upregulated significantly in breast cancer based on the results of the Oncomine and TCGA database analyses. Furthermore, the RT-qPCR outcome verified the high expression of MAL2 in breast cancer, which coincides with the results of the bioinformatics assay and previous studies in other tumors. Moreover, MAL2 expression was correlated with the prognosis of breast cancer. MAL2 could be a potential biomarker for breast cancer.

In clinical factors' analysis, MAL2 expression was found to be associated with vital status, ER, PR status, and *M* classification. The MAL2 level of dead patients was higher than that of alive patients, implying that patients with high MAL2 expression were more aggressive. Additionally, patients without metastasis had a higher MAL2 expression level than patients with metastasis, and high MAL2 expression was associated with ER- and HER2-positive patients.

Both the chi-square test and multivariate analysis showed that MAL2 expression is associated with stage and *M* classification. In our study, breast cancer patients with high expression had shorter overall survival. In the multivariate model, stage, *M* classification, and MAL2 expression were significantly related to OS (all *P* < 0.05), especially high MAL2 expression, advanced breast cancer patients, and those with distant metastasis. This finding may serve as a basis for the proper selection of specific and personalized treatment for breast cancer. Moreover, we found that MAL2 was an independent prognostic factor and might become a biomarker for breast cancer.

To identify the biological function of MAL2 in breast cancer, we used GSEA analysis to predict the pathway associated with MAL2. MYC target V1, mTORC1 signaling pathway, and E2F targets were closely related to the progression of breast cancer.

The high-MAL2-expression phenotype was associated with the activated MYC targets v1 and E2F targets v1 gene sets based on the enrichment score. MYC, a prominent gene in MYC targets v1 gene set, is common in aggressive tumors and contributes to cancer development [27, 28]. Also, MYC gene is overexpressed in triple-negative breast cancer and targeting the gene provides a new treatment [29]. Schulze's study showed that high MYC Targets v1 enrichment scores were associated with high mutation load, increased infiltration of pro-and anticancerous immune cells, tumor aggressiveness, and poor prognosis of ER-positive cancer [30].

Besides, the E2F transcription factors are downstream effectors of the retinoblastoma protein (pRB) pathway, which is essential to regulate numerous genes essential for DNA replication and cell cycle progression [31]. A previous study showed that E2F transcription factors played key roles in mediating tumor development and metastasis for knockout E2F, leading to the decreased tumor angiogenesis and metastatic capacity of breast cancer. E2Fs could control the expression of genes critical to angiogenesis, remodeling the extracellular matrix, tumor cell survival, and tumor cell interactions with vascular endothelial cells that boost breast cancer metastasis to the lungs [32]. Furthermore, the E2F enrichment score is a marker of breast cancer aggressiveness and predicts the responsiveness of ER-positive/HER2-negative patients to neoadjuvant chemotherapy [33]. Similar research also found that ER-dependent E2F transcription enhanced endocrine resistance in breast cancer [34]. Taken together, speculated high MAL2 expression might be associated with proliferation, metastasis, and prognosis of breast cancer by regulating the genes in these two gene sets.

We finally identified the relationship between the infiltrating level of immune cells and MAL2 using ssGSEA. Interestingly, MAL2 expression was found to be significantly associated with the negatively infiltrating level of eosinophils and plasmacytoid dendritic cells.

A prior study showed eosinophil infiltration was considered a favorable prognosis in breast cancer [35]. In addition, a previous study indicated that low baseline eosinophil count was related to a higher recurrence rate in 419 patients diagnosed with breast cancer [36]. Similar results could be found in recent studies. Low blood eosinophilic relative count was associated with a worse prognosis in 930 breast cancer patients [37]. Moreover, there was a positive correlation between eosinophilic relative counts and both pathological complete remission and survival rate in triple-negative and hormone receptor-negative/HER2-positive breast cancer patients [38].

As for plasmacytoid dendritic cells, it was reported that the proportion of plasmacytoid dendritic cells in triple-negative breast cancer was higher than other subtypes of breast cancer [39]. Besides, high plasmacytoid dendritic cells in triple-negative breast cancer were bound up with a favorable immune response and predicted better survival in 2968 breast cancer patients [39]. Interestingly, in the present study, we found the overexpression of MAL2 was significantly associated with the low infiltrating level of eosinophilic relative counts and plasmacytoid dendritic cells. Interestingly, in the present study, we found that the high MAL2 expression level had a significantly negative correlation with the infiltrating level of eosinophils and plasmacytoid dendritic cells. Moreover, high MAL2 expression was associated with poor OS in patients with breast cancer. Since high eosinophils and plasmacytoid dendritic cells predicted better breast cancer survival in previous research, we speculate that high‐expressed MAL2 might impact the eosinophils and plasmacytoid dendritic cells, which triggered a disadvantageous immune response, leading to poor prognosis in breast cancer.

To the best of our knowledge, this is the first research further analyzing the relationship between MAL2 expression and clinical features and immune infiltration. MAL2 expression may be an independent predictor of a poor disease survival prognosis in breast cancer patients. However, there are a few shortcomings in this study. The sample size included in the experimental validation part was small. And the collected breast cancer tissues were fresh samples, which could not be followed up according to the prognosis of the patients. Besides, further studies are needed to explore the mechanism of MAL2 in breast cancer.

## 5. Conclusion

In conclusion, our study showed MAL2 expression increased markedly in breast cancer patients and was related to overall survival. MAL2 might be a novel prognostic biomarker of breast cancer. Moreover, high MAL2 expression correlates with reduced immune infiltration of eosinophils and plasmacytoid dendritic cells in breast cancer. However, further studies are warranted to verify the value of MAL2 in breast cancer prognosis evaluation.

## Figures and Tables

**Table 1:** Details of MAL2 across six public expression data sets in the Oncomine database.

Data sets (sample size)	Comparison groups	Fold change	*P* value	Overexpression gene rank
Perou breast (65)	Ductal breast carcinoma vs. normal	3.586	1.59E–11	8 (in top 1%)
Sorlie breast (85)	Ductal breast carcinoma vs. normal	3.480	9.61E–9	31 (in top 1%)
Sorlie breast 2 (167)	Ductal breast carcinoma vs. normal	3.326	5.89E–5	148 (in top 3%)
Ma breast 4 (66)	Ductal breast carcinoma in situ epithelia vs. normal	2.417	5.26E–5	266 (in top 2%)
Curtis breast (2136)	Invasive ductal and invasive lobular breast carcinoma vs. normal	2.286	1.51E–27	432 (in top 3%)
	Tubular breast carcinoma vs. normal	2.093	1.80E–20	852 (in top 5%)
	Invasive ductal breast carcinoma vs. normal	2.168	5.89E–45	1803 (in top 10%)
TCGA breast (593)	Invasive lobular breast carcinoma vs. normal	2.437	1.35E–12	521 (in top 3%)
	Invasive breast carcinoma vs. normal	2.317	2.15E–12	1744 (in top 9%)
	Invasive ductal breast carcinoma vs. normal	2.403	1.29E–18	1851 (in top 10%)

**Table 2:** Clinical characteristics of TCGA breast cancer cohort.

Characteristics	Number of sample size (%)
Age	
<40	75 (6.88)
≥40	1015 (93.12)

Gender	
Female	1079 (98.90)
Male	12 (1.10)

Stage	
I	182 (16.70)
II	620 (56.88)
III	249 (22.84)
IV	21 (1.93)
X	18 (1.65)

*T* classification	
*T*1	280 (25.69)
*T*2	630 (57.80)
*T*3	138 (12.66)
*T*4	39 (3.58)
TX	3 (0.28)

*M* classification	
*M*0	900 (82.57)
*M*1	22 (2.02)
MX	168 (15.41)

*N* classification	
*N*0	484 (44.40)
*N*1	390 (35.78)
*N*2	180 (16.51)
*N*3	27 (2.48)
NX	9 (0.83)

ER status	
Negative	236 (21.65)
Positive	805 (73.85)
Unknown	49 (4.50)
PR status	343 (31.47)
Negative	695 (63.76)
Positive	52 (4.77)
Unknown	559 (51.28)
HER2 status	163 (14.95)
Negative	368 (33.76)
Positive	236 (21.65)
Unknown	805 (73.85)
Vital status	
Living	941 (86.33)
Deceased	149 (13.67)

MAL2 expression	
High	545 (50.00)
Low	545 (50.00)

NA, not available.

**Table 3:** Correlation between MAL2 mRNA expression and clinicopathologic parameters of breast cancer.

Parameters	Variables	N	MAL2 mRNA expression				*χ* ^2^	*P* value
			High	(%)	Low	(%)		
Age	<40	75	37	6.79	38	6.97	0	1
	≥40	1015	508	93.21	507	93.03		

Stage^*∗*^	I + II	802	392	73.82	410	75.79	—	**0.044**
	III + IV	270	139	26.18	131	24.21		
	X	18	14	2.57	4	0.73		

*T* classification^*∗*^	*T*1 + T2	910	466	85.82	444	81.62	—	0.115
	*T*3 + T4	177	77	14.18	100	18.38		
	TX	3	2	0.37	1	0.18		

*M* classification	M0	900	472	86.61	428	78.53	12.83	**0.002**
	M1	22	10	1.83	12	2.20		
	MX	168	63	11.56	105	19.27		

*N* classification^*∗*^	*N*0	484	233	43.15	251	46.4	—	0.399
	*N*1 + *N*2 + *N*3	597	307	56.85	290	53.6		
	NX	9	5	0.92	4	0.73		

ER status	Negative	236	103	19.92	133	25.38	5.47	0.065
	Positive	805	414	80.08	391	74.62		
	Unknown	49	28	5.14	21	3.85		

PR status	Negative	343	166	32.11	177	33.97	0.73	0.694
	Positive	695	351	67.89	344	66.03		
	Unknown	52	28	5.14	24	4.4		

HER2 status	Negative	559	282	76.01	277	78.92	2.51	0.285
	Positive	163	89	23.99	74	21.08		
	Unknown	368	174	31.93	194	35.6		

Vital status	Living	941	453	83.12	488	89.54	8.99	**0.003**
	Deceased	149	92	16.88	57	10.46		

ER, estrogen receptor; HER2, human epidermal growth factor receptor-2; PR, progesterone receptor. Note. Bold values indicate statistically significant *P* < 0.05. ^*∗*^means Fisher's test.

**Table 4:** Univariate and multivariate analyses of overall survival in breast cancer.

Parameter	Univariate analysis			Multivariate analysis		
	Hazard ratio	95% CI	*P* value	Hazard ratio	95% CI	*P* value
Age (≥40/<40)	1.564	0.568	0.387	1.871	0.672	0.230
		–4.306			–5.205	
Stage (III + IV/I + II)	1.745	1.376–2.212	**<0.001**	1.473	1.032	**0.033**
					—	
					2.102	
*T* classification (*T*3 + *T*4/*T*1 + *T*2)	1.595	1.220–2.084	**0.001**	1.394	0.993–1.956	0.055
*N* classification (*N*1 + *N*2 + *N*3/*N*0)	1.675	1.023–2.742	**0.040**	0.992	0.542–1.816	0.979
*M* classification (*M*1/*M*0)	7.873	3.378–18.351	**<0.001**	3.093	1.215–7.878	**0.018**
Estrogen receptor (positive/negative)	0.612	0.369–1.015	0.057	0.506	0.220–1.160	0.107
Progesterone receptor (positive/negative)	0.748	0.465–1.205	0.233	0.932	0.425–2.047	0.862
HER2 (positive/negative)	1.553	0.914–2.638	0.104	1.256	0.723–2.180	0.419
MAL2 (high/low)	1.626	1.012–2.612	**0.045**	1.792	1.093–2.937	**0.021**

**Table 5:** Gene sets enriched in high-MAL2-expression phenotype.

MSigDB collection	Name	ES	NES	NOM *P* value	FDR q-value
h.all. v7.4.symbols.gmt	HALLMARK_MYC_TARGETS_V1	0.809	2.089	<0.001	0.002
	HALLMARK_MTORC1_SIGNALING	0.734	2.012	0.002	0.007
	HALLMARK_E2F_TARGETS	0.866	1.964	0.000	0.011
	HALLMARK_MITOTIC_SPINDLE	0.751	1.948	0.004	0.010
	HALLMARK_G2M_CHECKPOINT	0.609	1.941	0.010	0.010
	HALLMARK_UV_RESPONSE_UP	0.618	1.724	0.018	0.049
	HALLMARK_PEROXISOME	0.545	1.581	0.035	0.113

c2.cp.kegg.v7.4.symbols.gmt	KEGG_OOCYTE_MEIOSIS	0.728	2.039	<0.001	0.011
	KEGG_SPLICEOSOME	0.755	1.974	0.004	0.011
	KEGG_CELL_CYCLE	0.785	1.940	0.002	0.010
	KEGG_INSULIN_SIGNALING_PATHWAY	0.574	1.725	0.015	0.051
	KEGG_UBIQUITIN_MEDIATED_PROTEOLYSIS	0.575	1.681	0.047	0.055

FDR, false discovery rate; ES, enrichment score; NES, normalized enrichment score; NOM, nominal. Notes: |NES| > 1, FDR q-value < 0.25, and NOM-*P* value < 0.05 were considered significantly different.

## Data Availability

Publicly available datasets were analyzed in this study. The data can be found at TCGA repository, https://www.cancer.gov/tcga, and Oncomine repository, http://www.oncomine.org. The data used to support the findings are available from the corresponding author upon request.

## References

[1] Bray F., Ferlay J., Soerjomataram I., Siegel R. L., Torre L. A., Jemal A. (2018). Global cancer statistics 2018: GLOBOCAN estimates of incidence and mortality worldwide for 36 cancers in 185 countries. *CA: A Cancer Journal for Clinicians*.

[2] Kasiappan R., Rajarajan D. (2017). Role of MicroRNA regulation in obesity-associated breast cancer: nutritional perspectives. *Advances in Nutrition: An International Review Journal*.

[3] Miller K. D., Nogueira L., Mariotto A. B. (2019). Cancer treatment and survivorship statistics, 2019. *CA: A Cancer Journal for Clinicians*.

[4] Huang R., Chen Z., He L. (2017). Mass spectrometry-assisted gel-based proteomics in cancer biomarker discovery: approaches and application. *Theranostics*.

[5] Duffy M. J., Walsh S., McDermott E. W., Crown J. (2015). Biomarkers in breast cancer. *Advances in Clinical Chemistry*.

[6] Byrne J. A., Maleki S., Hardy J. R. (2010). MAL2 and tumor protein D52 (TPD52) are frequently overexpressed in ovarian carcinoma, but differentially associated with histological subtype and patient outcome. *BMC Cancer*.

[7] Wilson S. H., Bailey A. M., Nourse C. R., Mattei M. G., Byrne J. A. (2001). Identification of MAL2, a novel member of the mal proteolipid family, though interactions with TPD52-like proteins in the yeast two-hybrid system. *Genomics*.

[8] Eguchi D., Ohuchida K., Kozono S. (2013). MAL2 expression predicts distant metastasis and short survival in pancreatic cancer. *Surgery*.

[9] Gao X., Chen Z., Li A., Zhang X., Cai X. (2018). MiR-129 regulates growth and invasion by targeting MAL2 in papillary thyroid carcinoma. *Biomedicine & Pharmacotherapy*.

[10] Shrout J., Yousefzadeh M., Dodd A. (2008). *β*2microglobulin mRNA expression levels are prognostic for lymph node metastasis in colorectal cancer patients. *British Journal of Cancer*.

[11] Fang Y., Wang L., Wan C. (2021). MAL2 drives immune evasion in breast cancer by suppressing tumor antigen presentation. *Journal of Clinical Investigation*.

[12] Bhandari A., Shen Y., Sindan N. (2018). MAL2 promotes proliferation, migration, and invasion through regulating epithelial-mesenchymal transition in breast cancer cell lines. *Biochemical and Biophysical Research Communications*.

[13] Shehata M., Bièche I., Boutros R. (2008). Nonredundant functions for tumor protein D52-like proteins support specific targeting of TPD52. *Clinical Cancer Research*.

[14] Rhodes D. R., Kalyana-Sundaram S., Mahavisno V. (2007). Oncomine 3.0: genes, pathways, and networks in a collection of 18,000 cancer gene expression profiles. *Neoplasia*.

[15] Rhodes D. R., Yu J., Shanker K. (2004). ONCOMINE: a cancer microarray database and integrated data-mining platform. *Neoplasia*.

[16] R. C. T. (2020). *R: A Language and Environment for Statistical Computing*.

[17] Ginestet C. (2011). ggplot2: elegant graphics for data analysis. *Journal of the Royal Statistical Society: Series A*.

[18] Jiao Y., Li Y., Lu Z., Liu Y. (2018). High trophinin-associated protein expression is an independent predictor of poor survival in liver cancer. *Digestive Diseases and Sciences*.

[19] Li Q., Ma W., Chen S. (2020). High integrin *α*3 expression is associated with poor prognosis in patients with non-small cell lung cancer. *Translational Lung Cancer Research*.

[20] Zhao E., Zhou C., Chen S. (2021). Flap endonuclease 1 (FEN1) as a novel diagnostic and prognostic biomarker for gastric cancer. *Clinics and Research in Hepatology and Gastroenterology*.

[21] Sun Z., Sun L., He M., Pang Y., Yang Z., Wang J. (2019). Low BCL7A expression predicts poor prognosis in ovarian cancer. *Journal of Ovarian Research*.

[22] Subramanian A., Tamayo P., Mootha V. K. (2005). Gene set enrichment analysis: a knowledge-based approach for interpreting genome-wide expression profiles. *Proceedings of the National Academy of Sciences*.

[23] Goldman M. J., Craft B., Hastie M. (2020). Visualizing and interpreting cancer genomics data via the Xena platform. *Nature Biotechnology*.

[24] Charoentong P., Finotello F., Angelova M. (2017). Pan-cancer immunogenomic analyses reveal genotype-immunophenotype relationships and predictors of response to checkpoint blockade. *Cell Reports*.

[25] Hänzelmann S., Castelo R., Guinney J. (2013). GSVA: gene set variation analysis for microarray and RNA-seq data. *BMC Bioinformatics*.

[26] Chen Y., Zheng B., Robbins D. H. (2007). Accurate discrimination of pancreatic ductal adenocarcinoma and chronic pancreatitis using multimarker expression data and samples obtained by minimally invasive fine needle aspiration. *International Journal of Cancer*.

[27] Sodir N. M., Kortlever R. M., Barthet V. J. A. (2020). MYC instructs and maintains pancreatic adenocarcinoma phenotype. *Cancer Discovery*.

[28] Lawson D. A., Bhakta N. R., Kessenbrock K. (2015). Single-cell analysis reveals a stem-cell program in human metastatic breast cancer cells. *Nature*.

[29] Camarda R., Zhou A. Y., Kohnz R. A. (2016). Inhibition of fatty acid oxidation as a therapy for MYC-overexpressing triple-negative breast cancer. *Nature Medicine*.

[30] Schulze A., Oshi M., Endo I., Takabe K. (2020). MYC targets scores are associated with cancer aggressiveness and poor survival in ER-positive primary and metastatic breast cancer. *International Journal of Molecular Sciences*.

[31] Hollern D. P., Honeysett J., Cardiff R. D., Andrechek E. R. (2014). The E2F transcription factors regulate tumor development and metastasis in a mouse model of metastatic breast cancer. *Molecular and Cellular Biology*.

[32] Bracken A. P., Ciro M., Cocito A., Helin K. (2004). E2F target genes: unraveling the biology. *Trends in Biochemical Sciences*.

[33] Oshi M., Takahashi H., Tokumaru Y. (2020). The E2F pathway score as a predictive biomarker of response to neoadjuvant therapy in ER+/HER2- breast cancer. *Cells*.

[34] Miller T. W., Balko J. M., Fox E. M. (2011). Er*α*-dependent E2F transcription can mediate resistance to estrogen deprivation in human breast cancer. *Cancer Discovery*.

[35] Sakkal S., Miller S., Apostolopoulos V., Nurgali K. (2016). Eosinophils in cancer: favourable or unfavourable?. *Current Medicinal Chemistry*.

[36] Ownby H. E., Roi L. D., Isenberg R. R., Brennan M. J. (1983). Peripheral lymphocyte and eosinophil counts as indicators of prognosis in primary breast cancer. *Cancer*.

[37] Onesti C. E., Josse C., Boulet D. (2020). Blood eosinophilic relative count is prognostic for breast cancer and associated with the presence of tumor at diagnosis and at time of relapse. *OncoImmunology*.

[38] Onesti C. E., Josse C., Poncin A. (2018). Predictive and prognostic role of peripheral blood eosinophil count in triple-negative and hormone receptor-negative/HER2-positive breast cancer patients undergoing neoadjuvant treatment. *Oncotarget*.

[39] Oshi M., Newman S., Tokumaru Y. (2020). Plasmacytoid dendritic cell (pDC) infiltration correlate with tumor infiltrating lymphocytes, cancer immunity, and better survival in triple negative breast cancer (TNBC) more strongly than conventional dendritic cell (cDC). *Cancers*.

